# An Experimental Investigation of Twelve Concrete Beams Post-Tensioned with Unbonded Tendons Under Center-Point and Third-Point Loading

**DOI:** 10.3390/ma18184333

**Published:** 2025-09-16

**Authors:** Wojciech Politalski, Andrzej Seruga

**Affiliations:** 1 Faculty of Civil Engineering and Resource Management, AGH University of Krakow, A. Mickiewicza 30, 30-059 Krakow, Poland; 2 Faculty of Civil Engineering, Tadeusz Kościuszko Cracow University of Technology, 31-155 Krakow, Poland; andrzej.seruga@pk.edu.pl

**Keywords:** flexural capacity, bending resistance, prestressed concrete, tendon stress increase, ultimate stress, unbonded tendons

## Abstract

The first concrete structures post-tensioned with unbonded tendons were constructed in the 1950s. Despite the popularity of such a type of construction solution, the theory describing the behavior of members with unbonded prestress remains relatively unknown. Different standards, provisions, and theories described by scientists can be found in the literature. The main problem is related to determining the value of the prestressing force and its increments because it is dependent upon the member rather than the section due to a lack of bond between the concrete and the tendons. Both theoretical and experimental studies enable the definition of parameters that have an influence on stress increase. Three of the most important of these parameters were investigated in tests conducted by the authors. This paper presents the findings of an experimental study conducted on twelve simply supported RC beams that were prestressed with unbonded tendons. A total of twelve elements were grouped according to various criteria, including their span-to-depth ratio, prestressed reinforcement ratio, and type of loading. All beams had a low reinforcing bars index, which met the Eurocode 2 requirements. The aim of this research was to check if such a level of ordinary reinforcement ratio will enable the achievement of a satisfactory crack pattern and also a high stress increase in unbonded tendons. The members were tested to investigate their behavior and the stress increment in tendons in terms of their load-carrying capacity.

## 1. Introduction

For decades, both experimental and theoretical research has been conducted on members post-tensioned with unbonded tendons. There are several advantages that make this construction solution very popular, especially in multi-span structures. The primary benefit of this design is that it reduces the friction coefficient between tendons and ducts. This, in turn, minimizes the prestressing force losses when compared to structures that utilize bonded tendons. The other advantages are the possibility of the adoption of a smaller bending radius of ducts (particularly important in slab system); the necessity for expensive and time-consuming injections is eliminated; the addition of wax or grease to the tendon duct protects the prestressing steel against corrosion from the production phase, during transport, storage on the construction site, and throughout the lifetime of structure. The primary disadvantages are lower fire and fatigue resistance. The prestressing force is maintained exclusively by anchorage, as opposed to the bonded prestressing systems in which the anchorage and injection both contribute to its maintenance [1]. Due to a lack of bond detailing of anchorage, zones should be considered with a prestressing force that corresponds to the tensile strength of prestressing steel [2].

The main problem in bending resistance calculations of structures post-tensioned with unbonded tendons is the estimation of the prestressing force value at failure. This is due to the fact that the stress increase in tendons is dependent upon the member rather than the section. The equations below describe the stress value in prestressing steel:

- Using Eurocode 2 [3] notations:
  (1a) $$\sigma_{\mathrm{pmt}}=\sigma_{\mathrm{pm}\infty }+\Delta \sigma_{p,\mathrm{ULS}}$$
- Using ACI 318-25 [4] notations:
  (1b) $$f_{ps}=f_{\mathrm{pe}}+\Delta f_{ps}$$
  where:

*σ_pmt_* and *f_ps_*—stress in tendon at ultimate;
*σ_pm∞_* and *f_pe_*—effective prestress—stress in tendon after immediate and time-dependent losses;
*Δσ_p,ULS_* and *Δf_ps_*—stress increase in unbonded tendon beyond effective prestress.

The first code provisions were given in ACI 318-63 [5]. Due to the limited number of conducted tests and a lack of appropriate knowledge about the behavior of such a type of structure, the proposed value of *Δf_ps_* was 15,000 psi (105 MPa). The same recommendations can be found in the present version of EC2 [3]. The recommended value of *Δσ_p,ULS_* is 100 MPa.

Mattock, Yamazaki, and Kattula [6] proposed an equation that describes the stress increase in unbonded tendons. This equation was introduced in a more conservative version in ACI 318-71 [7]. The following equation took into consideration two important parameters: concrete compressive strength and prestressing reinforcement ratio.

(2) $$f_{ps}=f_{\mathrm{pe}}+70+\frac{{f^{\prime }}_{c}}{100\cdot \rho_{p}},[\mathrm{MPa}]$$

with the following restrictions: *f_ps_* ≤ *f_pe_* + 420 MPa; *f_ps_* ≤ *f_py_* and *f_pe_* ≥ 0.5 *f_pu_*.

where:

*ρ_p_*—reinforcement ratio for prestressing reinforcement;
*f_c_′*—compressive strength of concrete;
*f_py_*—specified yield strength of prestressing reinforcement;
*f_pu_*—specified tensile strength of prestressing reinforcement.

Subsequent laboratory tests conducted by Mojtahedi and Gamble [8] indicated that the above equation (Equation (2)) could overestimate values of the stress increment for members with a span-to-depth ratio equal to 45. This might lead to hazardous scenarios because real bending moment resistance could be smaller than the calculated value when employing the above-mentioned code equation. Consequently, an additional equation (Equation (3)) for elements with a span-to-depth ratio greater than 35 was introduced to ACI 318-83 [9]. Equation (2) is valid for members with a span-to-depth ratio smaller than 35.

(3) $$f_{ps}=f_{\mathrm{pe}}+70+\frac{{f^{\prime }}_{c}}{300\cdot \rho_{p}},[MPa]$$

with the following restrictions: *f_ps_* ≤ *f_pe_* + 200 MPa; *f_ps_* ≤ *f_py_* and *f_pe_* ≥ 0.5 *f_pu_*.

It is noteworthy that these two equations persist in their functionality within the most recent version of ACI 318-25 [4]. The stress increment in unbonded tendons described in the ACI Code is dependent upon three parameters: the compressive strength of concrete, the reinforcement ratio for prestressing reinforcement, and the span-to-depth ratio.

**Figure 1:**
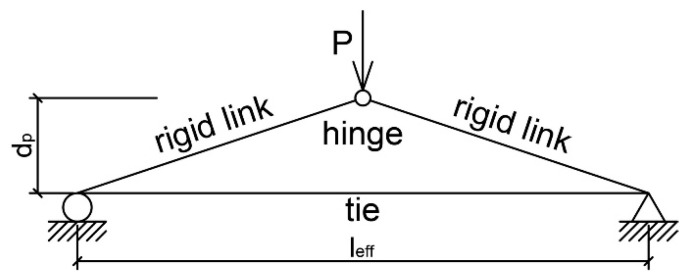
Truss model for members with unbonded tendons by Mojtahedi and Gamble [8].

**Figure 2:**
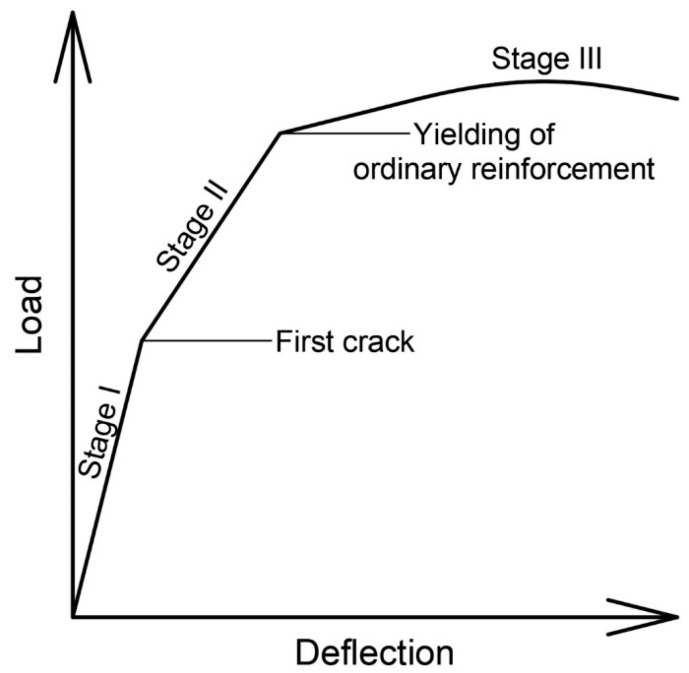
Simplified load–deflection curve for member with unbonded tendons [14].

In the literature, many articles can be found describing experimental research that provides knowledge about the behavior of members post-tensioned with unbonded tendons in comparison to members with bonded tendons. Some of the articles recognized by authors as the most important are listed below:

- Janney J., Hognestad E., McHenry D. [10] investigated 19 rectangular simply supported beams under third-point loading. Of these, three were pretensioned, three were post-tensioned with bonded tendons, five were post-tensioned with unbonded tendons without deformed bars, five were post-tensioned with unbonded tendons with deformed bars, and five were reinforced with deformed bars only. It is worth emphasizing that in the group with prestressing reinforcement without bond and without additional rebars, only one or two cracks occurred. They had significantly lower capacity than members post-tensioned with bonded tendons.
- Pannell F. N. [11] conducted research on 38 simply supported members with unbonded tendons. The following parameters were tested: stress in tendon after all losses *f_pe_*, prestressing reinforcement ratio, and span-to-depth ratio.
- Mattock A., Yamazaki J., Kattula B. [6] tested three two-span continuous T-beams, four simply supported T-beams, and three simply supported rectangular beams. They investigated members with both bonded and unbonded tendons and proved that stress increments in unbonded tendons could reach much greater values than the recommended 105 MPa. Their work prompted the introduction of changes in the ACI Code.
- Tam A., Pannell F. N. [12] performed tests on eight simply supported rectangular beams under center-point loading. They tested the influence of the following parameters on the behavior of members post-tensioned with unbonded tendons: stress in tendon after all losses *f_pe_*, reinforcement ratio for rebars and prestressing reinforcement, span-to-depth ratio, and compressive strength of concrete.
- Mojtahedi and Gamble [8] analyzed 16 beams with a large span-to-depth ratio. They indicated that the equation presented above (Equation (2)) can overestimate the stress increment in unbonded tendons and underestimate the flexural capacity of members. This happens due to the fact that only a few cracks appeared in the maximum moment region. Poor crack formation and propagation caused a small plastic hinge length, which led to more truss-like than beam-like behavior of the tested members (Figure 1).
- The aim of the test conducted by Cooke N., Park R., Yong Ph. [13] was to question the correctness of Equation (2). They tested nine simply supported members under third-point loading post-tensioned with unbonded tendons, divided into three groups with different span-to-depth ratios. The fourth group consisted of members with bonded tendons. The prestressing reinforcement ratio was the parameter that changed within each group.
- Du G., and Tao X. [14] investigated 26 simply supported members under third-point loading. Of these, 22 were with unbonded tendons and 4 were with bonded tendons. They all had the same span-to-depth ratio and differed with regard to concrete compressive strength, deformation of the bars, and the prestressing steel ratios. It was noted that the introduction of the proper amount of bar deformation led to better behavior of members post-tensioned with unbonded tendons. They acted in a similar way to those with bonded tendons. They described three phases of the response of elements to acting loading (Figure 2). Stage I is linear elastic uncracked, stage II is linear elastic cracked, and finally, stage III is the plastic phase. In members with low and medium values of the mechanical reinforcement ratio, all three phases could be observed, while for elements with a high value, only the first two were. They proposed boundary values of the reinforcement index, which can support the prediction of the behavior of the members.
- Campbell T. I. and Chouinard K. L. [15] tested six simply supported elements. They had the same span-to-depth ratio and prestressing reinforcement ratio. The only parameter that was changed during the experiment was the number of rebars.
- Research conducted by Harajli M. and Kanj M. [16] on 26 beams considered the following parameters: span-to-depth ratio (20, 13, and 8), type of loading (center-point and third-point), deformed and prestressing reinforcement ratio, and concrete compressive strength. A few of the members did not meet the ACI Code requirement regarding the minimum amount of ordinary reinforcement. In those under center-point loading, the behavior was not satisfactory due to the appearance of a large crack in the maximum moment region. In those under third-point loading, a few cracks in the region of constant moment appeared, but only one or two propagated at the final stage of loading.
- Chakrabarti P. et al. [17,18,19] tested 33 both rectangular and T-beam members. They tested the following parameters: deformed and prestressing reinforcement ratio, type of cross-section, concrete compressive strength, span-to-depth ratio, and stress in tendon after all losses.
- Daher Y. M. [20] investigated 11 simply supported beams—7 and 4 made from regular and high-strength concrete, respectively. The main aim was to establish the minimum reinforcement ratio, which would provide satisfactory behavior of the member up until the point of failure. All beams had the same cross-sectional dimensions and span-to-depth ratio. Both deformed and prestressing reinforcement ratios varied.
- Nassif H. et al. [21,22,23] tested 25 members—9 rectangular beams and 16 T-beams—under third-point loading. The following parameters were investigated: span-to-depth ratio, deformed and prestressing reinforcement ratio, stress in tendon after all losses, concrete compressive strength (HSC), and the type of cross-section. In most of the elements, three phases (elastic uncracked, elastic cracked, and plastic) were observed.
- Zhou W. and Zheng W. [24] conducted research on 16 two-span continuous beams with the same cross-section and concrete compressive strength under mid-span loading. They observed six stages during testing: stage I—elastic phase; stage II—first cracking at middle support; stage III—first cracking at span; stage IV—plastic hinge at middle support; stage V—plastic hinge at span, and stage VI—failure. During the first three stages, the stress increment in unbonded tendons was low and increased linearly. During the last three stages, the stress increment suddenly increased.
- Maguire M. et al. [25] tested four two-span slab specimens with span–depth ratios above 30. In the first two cases, the loading was acting in the live-end span and dead-end span, respectively, inducing maximum positive bending moments. In the two remaining specimens, the live load was acting in both spans, inducing an elastic maximum negative bending moment at the support and forming the first hinge at the center support and secondary hinges in both spans. Tendon stress increases in the tendons were compared to prediction equations by AASHTO LRFD, ACI 318 Building Code, Naaman, and Alkhairi. The test results and calculations indicated measured-to-predicted ratios between 1.23 and 3.99 and showed large scatter.
- Nakari T et al. [26,27] conducted research on four two-span T-shaped beams. All beams had the same number of unbonded tendons, and the degree of prestressing was kept constant. By contrast, the amount of passive reinforcement at the center support varied. Rotation capacity and moment redistribution were tested. The stress increments in unbonded tendons were considerably large in all tested members and allowed them to reach the yield point of tendon reinforcement.

A preliminary step in the analysis of the aforementioned tests is the introduction of the mechanical reinforcement ratios (Equations (4)–(7)). These coefficients connect important material and geometric characteristics of members, such as cross-sectional area *A_c_*, compressive strength of concrete *f_c_*′, area of reinforcement—both deformed bars *A_s_* and tendons *A_p_*, yield strength *f_y_*, effective prestress in unbonded tendons prior to loading *f_pe_*, and ultimate stress in unbonded tendons at failure *f_ps_*. Some authors use a prestressing steel index related to *f_pe_*, while others to *f_ps_*. This causes some problems in the interpretation of results.

(4) qs=AsfyAcf′c

(5) qe=ApfpeAcf′c

(6) $$q_{0}=q_{s}+q_{e}$$

(7) qu=AsfyAcf′+ApfpsAcf′c

The results gained from the above experiments can be divided into two groups of elements.

The first group is related to members without ordinary reinforcement (*q_s_* = 0) and the only reinforcements that are presented in the cross-section are unbonded tendons. Table 1 presents the range of factors achieved in the chosen tests. Depending on the prestressing steel index value *q_e_*, four kinds of member behaviors could be expected:

- At very low values of the *q_e_* index, the ultimate moment is equal to the cracking moment. After the appearance of the first crack, the rapid increase in deflection and the lack of member response could be expected. Such a behavior was observed in experiments conducted by Pannell (for *q_e_* ≤ 0.15), Cook et al. (UB9—*q_e_* = 0.032), and Daher (R-4—*q_e_* = 0.028).
- At low values of the q_e_ index, after the appearance of the first crack, a rapid increase in deflection and poor member response could be expected. The deflection increase led to stress increments in unbonded tendons, which allowed the achievement of bending resistance greater than the cracking moment. This kind of behavior was observed in tests performed by Cooke et al. (UB3 and UB6—*q_e_* = 0.033 and 0.029, respectively) and Daher (R-1 and RH-1—*q_e_* = 0.033 and 0.03, respectively).
- At average values of the q_e_ index, more cracks are expected than with previous q_e_ index values, but still fewer cracks than in pretensioned and post-tensioned members with a bond between the prestressing steel and the concrete. The occurrence of the first crack will not lead to a rapid deflection increment and a poorer response to external loading. Such behavior was observed in experiments conducted by Janney et al. (all elements from Group 3 except 3-.428), Pannell (for 0.15 < *q_e_* ≤ 0.30), Cooke et al. (UB2, UB5, and UB8—*q_e_* = 0.125, 0.111, and 0.125, respectively), Campbell et al. (beam no. 1—*q_e_* = 0.139), and Nassif (rectangular beam no. 8 and T-beam no. 13—*q_e_* = 0.064 and 0.030, respectively).
- At high values of the *q_e_* index, the crack pattern is expected to be very similar to those in pretensioned and post-tensioned members with a bond between the prestressing steel and the concrete, but due to a lack of bonded reinforcement, deviation of the crack width could be greater. This kind of behavior was observed in a test performed by Janney et al. (element 3-.428), Pannell (*q_e_* > 0.30), and Cooke et al. (UB1, UB4, and UB7—*q_e_* = 0.254, 0.223, and 0.249, respectively). It is worth emphasizing that the bending resistance of members UB4 and UB7 was slightly greater than member B4, which has the same properties but was post-tensioned with bonded tendons. This indicates that a high value of the *q_e_* index ensures similar behavior of these types of structures.

**Table 1:** Values of prestressing steel index in the chosen tests.

Team	Janney et al. [10]		Pannell [11]		Cooke et al. [13]		Daher [20]		Nassif et al. [21,22,23]	
**Index range**	q_e_ min	q_e_ max	q_e_ min	q_e_ max	q_e_ min	q_e_ max	q_e_ min	q_e_ max	q_e_ min	q_e_ max
	0.060	0.242	0.053	0.335	0.029	0.254	0.028	0.033	0.030	0.064

It seems that the prestressing steel index boundaries (0.15 and 0.3) proposed by Pannell are correct, but it should be noted that they are only approximate.

The second group of discussed members contained both ordinary reinforcement and unbonded tendons. Table 2 presents the range of factors achieved in the chosen tests. On the basis of the ordinary steel index value *q_s_* and the prestressing steel index value *q_e_*, the following observations could be made:

- At very low values of *q_s_* and q_0_ indexes, the member could behave in a similar manner to those with unbonded tendons only. After the appearance of one dominant crack, a sudden deflection and a lack of response to external loading could be expected. This kind of behavior was observed in a test conducted by Daher (R-2 and R-5—*q_s_* = 0.006 and RH-2 and RH-3, *q_s_* = 0.003 and 0.005, respectively).
- At low values of *q_s_* and *q*_0_ indexes, several cracks can occur in the initial phase of loading, but only one or two will propagate. This type of beam can achieve satisfying bending resistance and stress increments in unbonded tendons, but the crack pattern cannot be accepted due to the allowable crack width being exceeded. Such a behavior was noticed by Harajli (beams with *q_s_* ∈ <0.013–0.020> and *q*_0_ ∈ <0.039–0.135>).
- At an average value of the *q*_0_ index and a satisfactory value of *q_s_*, a similar behavior to that in members with bonded tendons can be expected. Proper crack formation and propagation are expected, and the load–deflection chart should include all three stages of member behavior: elastic uncracked, elastic cracked, and plastic. This statement was confirmed by the following tests: Janney et al. (elements 4-.131 and 4-.285—q_0_ = 0.097 and 0.184, respectively), Mattock et al. (T-beams TU1 and TU2—q_0_ = 0.054 and 0.044, respectively), Tam et al. (q_0_ < 0.2), Du et al. (q_0_ < 0.25), Campbell et al. (beams no. 2, 3, and 4—q_0_ = 0.213, 0.292, and 0.319, respectively), Chakrabarti et al. (*q_u_* ∈ <0.1 ÷ 0.25>), and Nassif (q_0_ < 0.25).
- With high values of the q_0_ index and a satisfactory value of q_s_, a similar behavior to that in members with bonded tendons can be expected. Proper crack formation and propagation is expected, and the load–deflection chart should include only the first two stages of member behavior: elastic uncracked and elastic cracked. Such behavior could be observed in elements tested by Janney et al. (element 4-.455—q_0_ = 0.270), Mattock et al. (rectangular beams RU1 and RU2—q_0_ = 0.299), Tam et al. (q_0_ > 0.2), Du et al. (q_0_ > 0.25), Campbell et al. (beams no. 5 and 6—q_0_ = 0.380, and 0.445, respectively), and Chakrabarti et al. (*q_u_* > 0.25).

**Table 2:** Values of reinforcing indexes in the chosen tests.

**Team**	**Janney et al.** [10]		**Mattock et al.** [6]		**Tam et al.** [12]		**Du et al.** [14]		**Campbell et al.** [15]	
**Index range**	q_s_ min	q_s_ max	q_s_ min	q_s_ max	q_s_ min	q_s_ max	q_s_ min	q_s_ max	q_s_ min	q_s_ max
	0.064	0.071	0.007	0.065	0.048	0.130	0.021	0.349	0.075	0.323
	q_0_ min	q_0_ max	q_0_ min	q_0_ max	q_0_ min	q_0_ max	q_0_ min	q_0_ max	q_0_ min	q_0_ max
	0.097	0.270	0.044	0.299	0.112	0.255	0.063	0.470	0.213	0.445
**Team**	**Harajli et al.** [16]		**Chakrabarti** [17,18,19]		**Daher** [20]		**Nassif et al.** [21,22,23]		**Zhou et al.** [24]	
**Index range**	q_s_ min	q_s_ max	q_s_ min	q_s_ max	q_s_ min	q_s_ max	q_s_ min	q_s_ max	q_s_ min	q_s_ max
	0.013	0.164	0.007	0.219	0.003	0.012	0.012	0.044	0.031	0.242
	q_0_ min	q_0_ max	q_0_ min	q_0_ max	q_0_ min	q_0_ max	q_0_ min	q_0_ max	q_0_ min	q_0_ max
	0.039	0.255	0.023	0.318	0.017	0.043	0.030	0.180	0.085	0.477

It seems that the minimum amount of ordinary reinforcement, providing proper behavior of members, is more connected to the ordinary reinforcement index *q_s_* than the ordinary reinforcement ratio *ρ_s_*. The boundary value between “plain concrete” and “reinforced concrete” with unbonded tendons could be established as *q_s_* = 0.02. The steel index boundaries *q*_0_ (0.15 and 0.25) proposed by Du et al. are correct, but it should, once again, be noted that they have only an indicative value.

The results gained from experimental tests were used by researchers to build analytical models for determining the stress increment in unbonded tendons and the flexural capacity of members. Based on a regression analysis of parameters such as the span-to-depth ratio, concrete compressive strength, ordinary and prestressing reinforcement indexes, and the type of loading, straightforward equations were developed. It is worth mentioning researchers such as Naaman A. et al. [28,29,30], Harajli M. et al. [31,32,33], Lee L. H., Moon J. H., Lim J. H., [34], Chakrabarti [19], and Peng F., Xue W. [35].

## 2. Experimental Program

The test program, which was a part of a thesis submitted for a PhD in structural engineering [36], was preceded by initial experiments conducted on six simply supported members in the laboratory of the Faculty of Civil Engineering of Cracow University of Technology. These experiments are partly described in references [37,38,39,40]. Based on the received data, parametric analysis was performed using FEM DIANA [41]. This analysis took the following parameters into consideration: concrete class (from C30/37 to C90/105), span-to-depth ratio (from 20 to 50), prestressing reinforcement ratio (0.29% and 0.58%), and type of loading (center-point and third-point loading). Finally, the decision was made to conduct the test on twelve simply supported prestressed beams made of class C45/55 concrete.

The research had two main objectives: the first was to show that it is possible to achieve stress levels in unbonded tendons beyond the values indicated in the EC2 [3] and ACI 318 [4] codes, while the second one was to check if the minimum ordinary reinforcement indicated by EC2 [3] would be sufficient to achieve a satisfactory crack pattern and avoid sudden failure caused by the propagation of one or a few cracks in the maximum bending moment area. Aforementioned experimental and theoretical studies have identified three main parameters that influence the stress increment in unbonded tendons. The following factors must be taken into consideration: the span-to-depth ratio, the reinforcement ratio, and the type of loading. Research has indicated that as the span-to-depth ratio and reinforcement ratio decrease, the stress increase in unbonded tendons grows. It was further noted that the stress increment is associated with the configuration of the bending moment diagram. Consequently, the maximum value is anticipated for third-point loading, the medium value for uniform loading, and the minimum value for one-point loading.

### 2.1. Members Geometry

The beams were divided into three groups, distinguished by their span lengths. The observed parameters in each group also included the type of loading and the number of tendons. The description of each member consisted of the symbol LxxTyFz, in which Lxx indicated the span length given in decimeters and xx could take on values equal to 36, 60, or 84, which corresponded to span lengths of 3.6, 6.0, or 8.4 m, respectively. Ty indicated the number of prestressing tendons, which could be 1 or 2. Fz indicated the type of loading, where z could take on values equal to 1 or 2, which corresponded to center-point and third-point loading, respectively. For instance, L60T2F1 represents a beam with a span length of 6.0 m, prestressed with two tendons, and tested under center-point loading.

**Figure 3:**
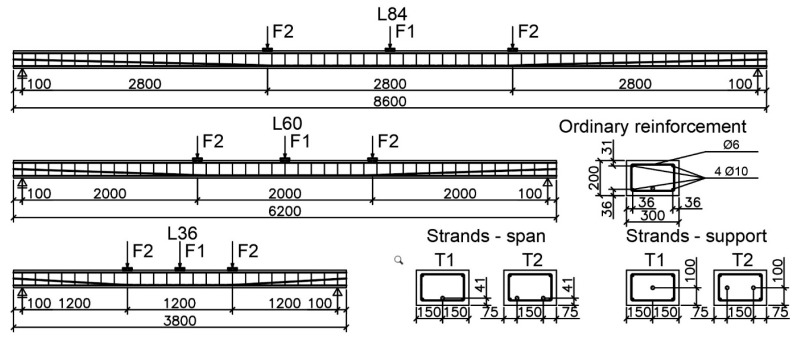
Side view and cross-sections of tested beams.

**Figure 4:**
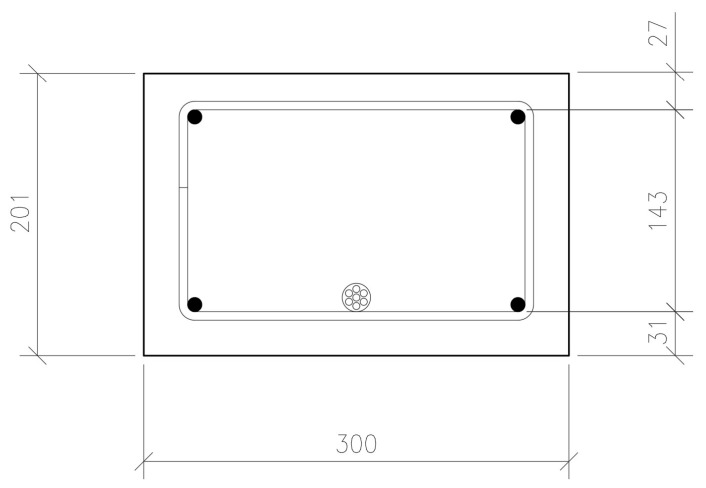
Mean values of cross-sectional dimensions.

The side views and cross-sections of all tested beams, in conjunction with the arrangement of reinforcing bars and prestressing tendons, are illustrated in Figure 3. The beams exhibited nominal cross-sections measuring 0.30 × 0.20 m^2^. Prior to the pouring of the concrete mix into the formwork, the dimensions of the cross-section and the reinforcement spacing were measured. These sections contained strain gauges and were located at one-third and one-half of the span length. The measured dimensions are presented in Table 3, and the average values are shown in Figure 4.

Longitudinal tensile and compressive reinforcement consisted of two 10 mm ribbed bars, each with a cross-sectional area of 157 mm^2^. The reinforcement ratio was approximately 0.33% and the mechanical reinforcement ratio was approximately 2.3%. Prestressing reinforcement consisted of one or two ø15.7 mm strands. The cross-sectional area of the tendon was 151 mm^2^. The prestressing reinforcement ratio was approximately 0.32% and 0.63%, and the mechanical prestressing reinforcement ratio was approximately 8.3% and 16.6% for one and two tendons, respectively. The shear reinforcement consisted of 6 mm two-legged stirrups with uniform spacing equal to 150 mm.

### 2.2. Material Properties

The ordered concrete mix needed to fulfil the requirements of concrete strength class C45/55, in accordance with EC2 [3]. Portland cement CEM I 42.5 R was used. The maximum aggregate size was specified as 16 mm. To gain a higher modulus of elasticity, basalt aggregate was supplied. The concrete cubes and cylinder samples were made at the time of casting and were kept in the same condition as the beams during curing. The first specimen was tested when the concrete was at an age of 31 days, and the last test was conducted at the age of 202 days. It could be observed that almost 4 months after casting, the concrete properties stabilized and did not significantly change up to the time when the last beam was tested. The concrete compressive cube strength was 81.30 MPa, and the concrete compressive cylinder strength was 66.82 MPa. The modulus of elasticity determined on the cylinder specimens was measured as 47.51 GPa. The Model Code 2010 [42] equation (Equation (8)) for calculating the modulus of elasticity for concrete based on basalt aggregate was used. The obtained result of 47.76 GPa had good convergence with the above-mentioned test result of 47.51 GPa.

(8) $$E_{ci}=E_{c0}\cdot \alpha_{E}\cdot {\left(\frac{f_{cm}}{10}\right)}^{1/3}$$

**Figure 5:**
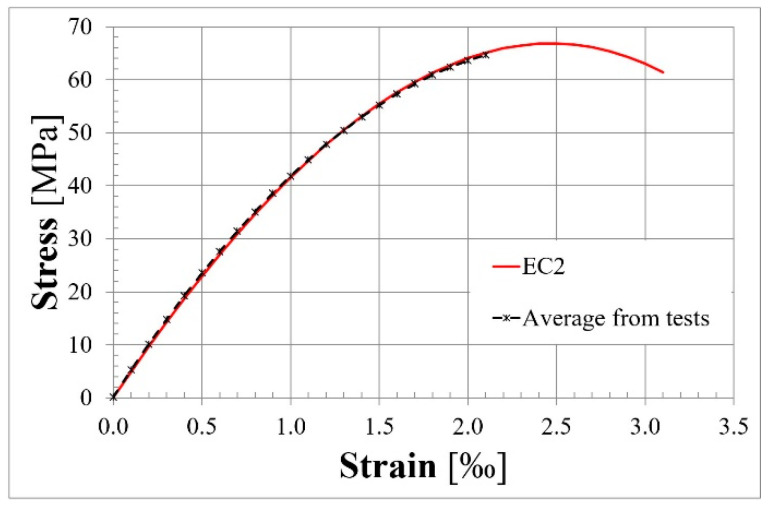
Stress–strain relationship for concrete.

The stress–strain relationship for concrete (Figure 5) also had good convergence with the equation below (Equation (9)), describing the non-linear relationship described in EC2 [3].

(9) $$\sigma_{c}=\frac{k\cdot \eta -\eta^{2}}{1+(k-2)\cdot \eta }\cdot f_{cm}$$

where

(10) $$k=1.05\cdot E_{cm}\cdot \frac{\varepsilon_{c1}}{f_{cm}}$$

(11) $$\eta =\frac{\varepsilon_{c}}{\varepsilon_{c1}}$$

The tensile strength of concrete was determined from both direct and indirect tests. The obtained tensile strength and fracture energy were 3.56 MPa and 177.6 N/m, respectively. The tensile strength from the splitting cube tests was 4.93 MPa.

The longitudinal reinforcement test was conducted on three 10 mm reinforcing bars. The mean measured yield strength, tensile strength, and modulus of elasticity were 460 MPa, 694 MPa, and 202 GPa, respectively.

The prestressing reinforcement test was conducted on three ø15.7 mm strands. The mean measured characteristic value of the 0.1% proof stress, characteristic value of the ultimate strength, and modulus of elasticity were 1742 MPa, 1863 MPa, and 185 GPa, respectively.

**Figure 6:**
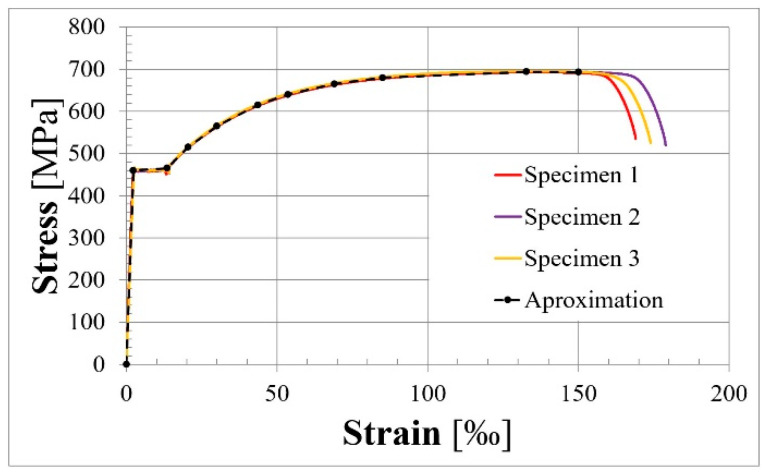
Stress–strain relationship for reinforcing bars.

**Figure 7:**
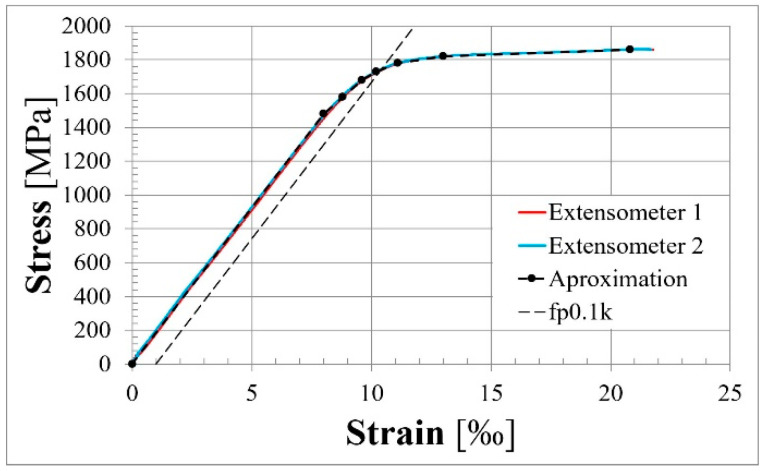
Stress–strain relationship for steel prestressing strands.

The obtained stress–strain relationships for rebars and tendons are presented in Figure 6 and Figure 7, respectively.

### 2.3. Test Stand and Instrumentation Equipment

**Figure 8:**
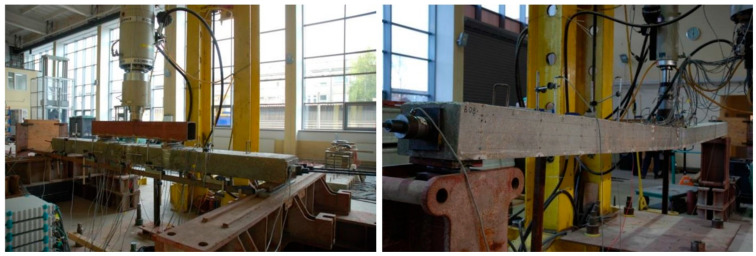
Test stands with beams during the test—Schenck 1000 (**left**) and Schenck 400 (**right**).

The test stand was located under a frame of the hydraulic test machine. The L36 members were tested with Schenck 1000, and members L60 and L84 were tested with Schenck 400. Third-point loading was realized with a traverse box section made of two channels welded toe to toe. The view of the stand is shown in Figure 8.

**Figure 9:**
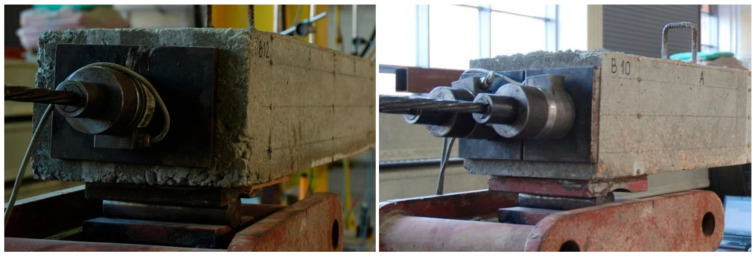
Force transducers arrangement.

**Figure 10:**
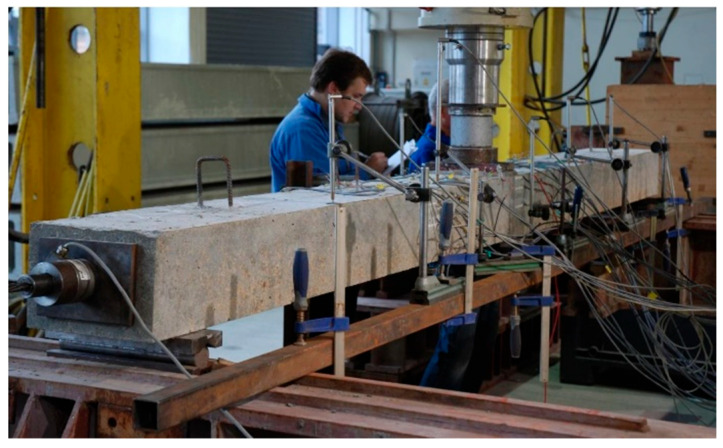
Displacement transducers arrangement.

**Figure 11:**
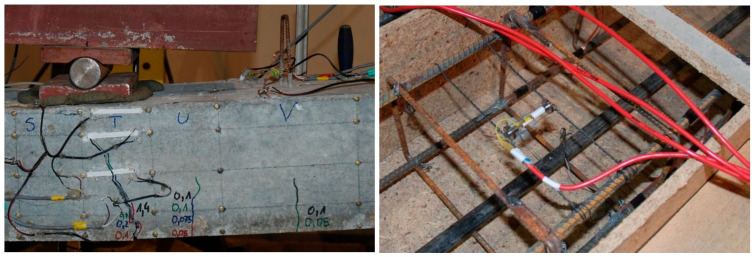
DEMEC discs grid and TC strain gauges (**left**); TS strain gauges and Geokon (**right**).

**Figure 12:**
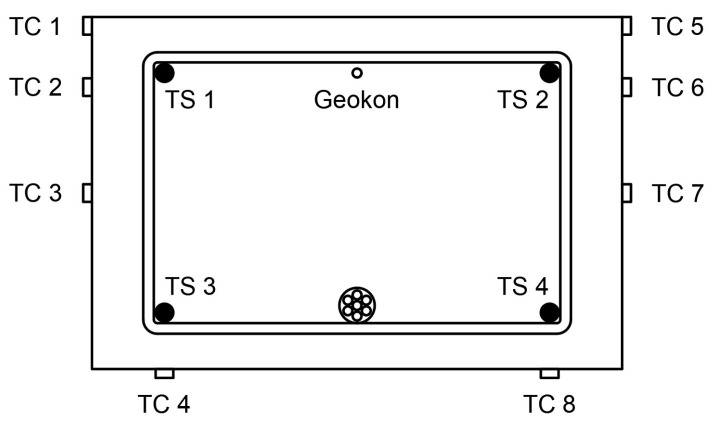
Strain gauge arrangement.

During the laboratory tests, the following measurements were taken:

- The loading force in the hydraulic test machine was recorded.
- Force in the unbonded tendons was measured by use of force transducers placed under each of the anchorages—two for T1 and four for T2 beams (Figure 9).
- Displacements at 1/6, 1/3, 1/2, 2/3, and 5/6 of the span length were measured by means of displacement transducers; the displacement in the mid-span was also controlled by a hydraulic machine piston extension (Figure 10).
- Strains along beams were measured using a detachable mechanical strain gauge DEMEC. Steel discs attached to the structure using a suitable adhesive were placed on the front face of the beam 5, 40, 100, 160, and 195 mm from the bottom (Figure 11).
- Strains in critical cross-sections (1/2 and 1/3 span lengths) were measured using electrical strain gauges. These were attached to the bottom, front, and rear faces of each beam, and also to the reinforcing bars. A sketch presenting the strain gauge arrangement is shown in Figure 12. In the mid-span of each beam, a Geokon vibrating wire strain gauge was placed. It was placed in the center of the beam at the level of the upper reinforcement bars.

### 2.4. Research Timetable and Loading Phases

The first member was tested 139 days after concreting. The preparation of the member for testing and a preliminary analysis of a previously tested member enabled the examination of approximately two members per week. The last beam was tested 184 days after concreting. The beams were prestressed for a few days before conducting the tests. This ensured that the prestressing force could stabilize, and the initial relaxation of tendons could occur.

The first beam tested was L36T2F2. Due to the fact that it had the smallest span-to-depth ratio and it was post-tensioned with two tendons, it had the greatest bending resistance. The task of the first member was to reveal the behavior during the test; thus, it consisted of the largest number of loading phases. As can be seen in Table 4, each member test could be divided into two parts: the occurrence of the first crack and member failure. The testing of the following members consisted of two loading phases before cracking. In the first phase, the force acting from the hydraulic test machine was about 50% of the predicted cracking force. The force in the second phase was increased up to the time when the first crack was observed. The next phases were conducted at increments until member failure. After each loading, the increase in concrete strain was measured using a DEMEC mechanical strain gauge. The duration of this process was several dozen minutes.

## 3. Experimental Results

**Figure 13:**
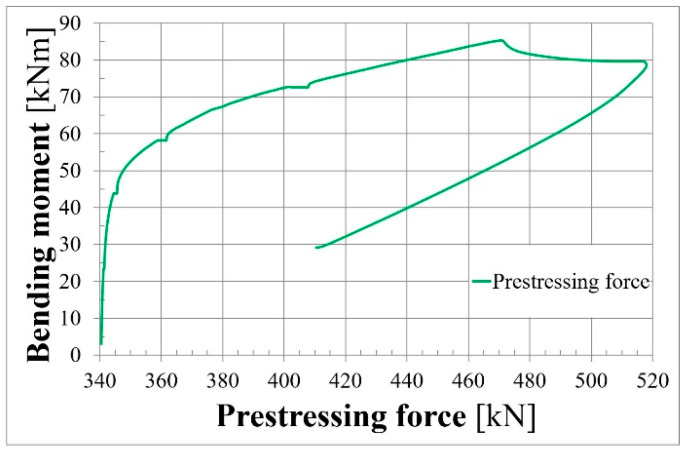
Prestressing force–bending moment relationship for L36T2F2.

**Figure 14:**
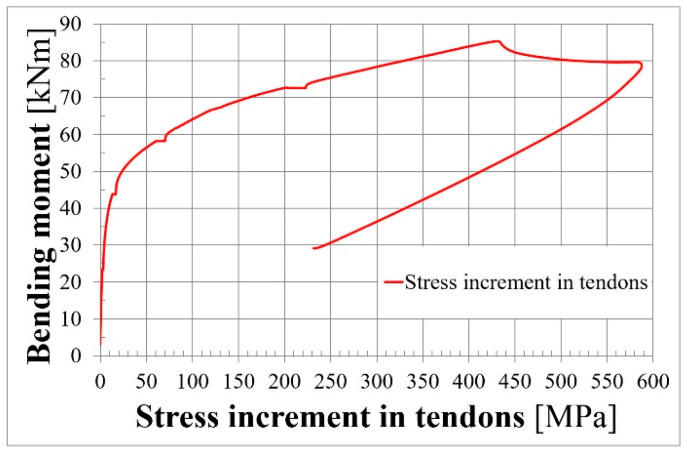
Stress increment–bending moment relationship for L36T2F2.

**Figure 15:**
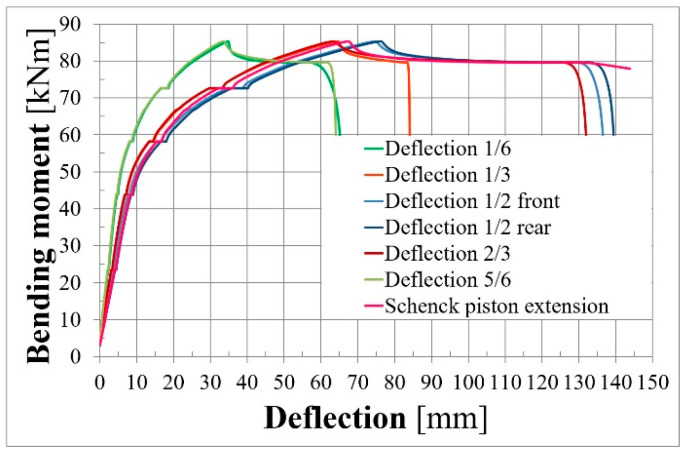
Deflection–bending moment relationship for L36T2F2.

**Figure 16:**
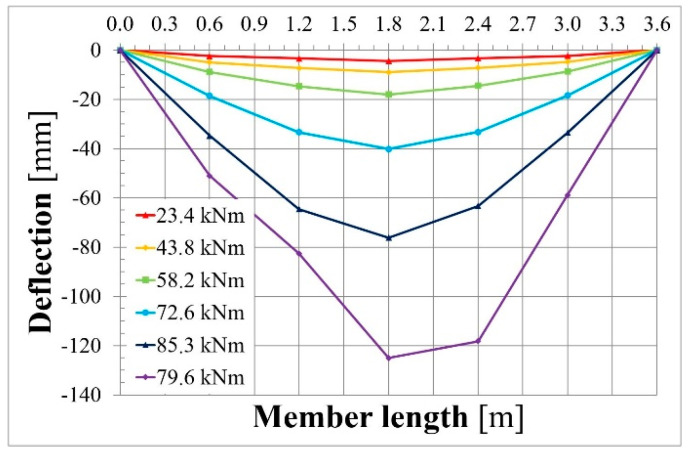
Span deflection of beam L36T2F2 in the chosen load phases.

**Figure 17:**
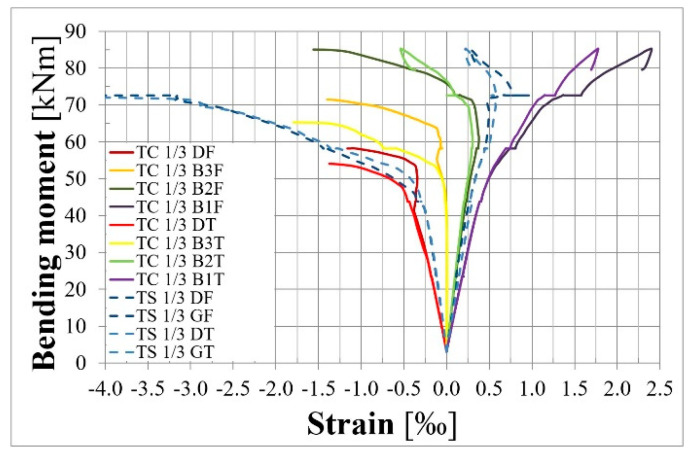
Strain–bending moment relationship in 1/3 of the span length of the L36T2F2 member.

**Figure 18:**
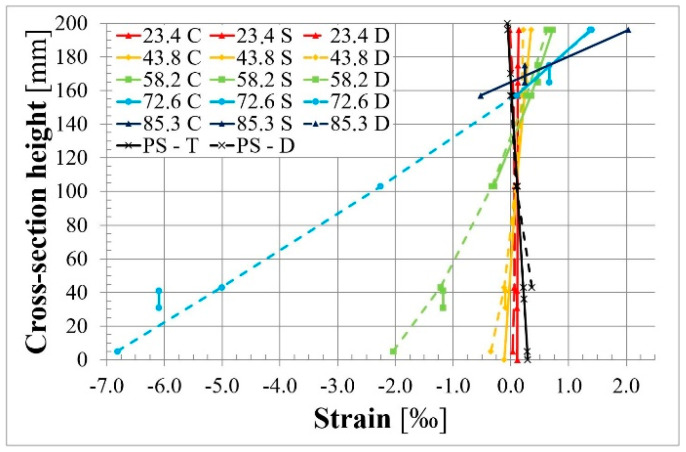
Strains in 1/3 of the span length of the L36T2F2 beam in the chosen load phases.

In the following section, detailed results for single member and the collation of results for all members are presented. The intention of the presentation manner shown in point 3.1 of this paper was to present the methodology employed in the collection of test results for each member. The chosen data presented in Figure 13, Figure 14, Figure 15, Figure 16, Figure 17 and Figure 18 has been gathered for all beams and is included in Table 5, Table 6, Table 7, Table 8 and Table 9.

### 3.1. Detailed Results for the L36T2F2 Member

Figure 13 and Figure 14 present prestressing force and stress increments, respectively, in two unbonded tendons.

Figure 15 presents the deflection–bending moment relationship for L36T2F2. Green lines are deflections for 1/6 and of the 5/6 span length. The orange and red lines show deflection at 1/3 and 2/3 of the span length, respectively. The blue lines present deflection measured by deflection gauges in the front and rear faces of the beam, and the pink line presents hydraulic machine piston extension. It can be observed that deflections in the symmetrical points of the beam are almost equal until reaching the bending capacity. Mid-span deflections measured in the front and rear faces of the beam also have the same values until reaching the bending capacity. This is caused by the fact that the beam cross-sectional width was greater than the height, and the beam was not vulnerable to torsion. It can be seen that the shapes of the deflection diagram (Figure 15) and the stress-increment diagram (Figure 14) are very similar.

Figure 16 presents deflection along the span length in the chosen loading phases. It can be observed that beam failure occurred in 1/3 of the span length.

Figure 17 presents strains measured in 1/3 of the span length. Electrical strain gauge symbols are described in Figure 12. The strains measured in rebars and concrete are presented using blue lines and other colors, respectively. Compressive strains in concrete are very similar in the front and rear faces of the member. Tensile strains in concrete differ due to the fact that the crack in this cross-section behaves slightly differently in the front and rear faces of the cross-section.

Figure 18 presents the strain distribution at the height of the cross–section in the chosen phases of loading. The black line presents strain due to prestressing; the solid and dashed lines are for theoretical calculations and values measured using DEMEC, respectively. Different colors of lines present strain in the chosen phases of loading. Solid lines show strain measured using electrical strain gauges (TC) in concrete. Dashed lines present the strain measured on the beam surface using the DEMEC mechanical strain gauge. Short 10 mm vertical lines denote the mean strain in steel reinforcing bars measured using electrical strain gauges (TS). It can be seen that strains at the same level of the cross-section are very similar in concrete and rebars, especially in the compressive zone. The small difference in the tensile zone could be caused by cracks in the concrete.

### 3.2. Compilation of Results for All Members

Table 5 presents reinforcement ratios and mechanical reinforcement ratios for both rebars and tendons. Mechanical reinforcement index for rebars had the same value of 2.3% for all members. This met the requirements for the minimum reinforcement ratio demanded by EC2 [3]. Literature studies indicate that adopting an index value greater than *q_s_* = 2% provides appropriate cracking behavior of members post-tensioned with unbonded tendons—several cracks of acceptable crack width instead of one or two cracks in the critical cross-section. Mechanical reinforcement ratio indexes q_0_ varied from 0.075 to 0.078 and from 0.128 to 0.130 for member groups T1 and T2, respectively. In both cases, these values were smaller than 0.150. Due to this fact, a high stress increase in unbonded tendons was achieved, as expected.

**Table 5:** Reinforcing bars and tendons indexes.

	Concrete		Ordinary Reinforcement		Prestressing Reinforcement			Indexes				
Name of Member	Modulus of Elasticity [GPa]	Compressive Strength [MPa]	Reinforcement Ratio ρ_s_ [-]	Yield Strength [MPa]	Reinforcement Ratio ρ_p_ [-]	Effective Prestress [MPa]	0.1% Proof Stress [MPa]	Ordinary Reinforcement Index *q_s_ = ρ_s_∙f_y_/f_c_*	Prestressing Reinforcement Index *q_e_ = ρ_p_∙f_pe_/f_c_*	Prestressing Reinforcement Index *q_ps_ = ρ_p_∙f_py_/f_c_*	Reinforcement Index *q_0_ = q_s_ + q_e_*	Reinforcement Index *q_y_ = q_s_ + q_ps_*
**L36T2F2**	47.5	66.8	0.33%	460	0.63%	1127	1742	0.023	0.107	0.165	0.130	0.188
**L36T2F1**	47.5	66.8	0.33%	460	0.63%	1109	1742	0.023	0.105	0.165	0.128	0.188
**L36T1F2**	47.5	66.8	0.33%	460	0.32%	1109	1742	0.023	0.053	0.083	0.075	0.105
**L36T1F1**	47.5	66.8	0.33%	460	0.32%	1138	1742	0.023	0.054	0.083	0.077	0.105
**L60T2F2**	47.5	66.8	0.33%	460	0.63%	1127	1742	0.023	0.107	0.165	0.130	0.188
**L60T2F1**	47.5	66.8	0.33%	460	0.63%	1136	1742	0.023	0.108	0.165	0.130	0.188
**L60T1F2**	47.5	66.8	0.33%	460	0.32%	1130	1742	0.023	0.054	0.083	0.076	0.105
**L60T1F1**	47.5	66.8	0.33%	460	0.32%	1135	1742	0.023	0.054	0.083	0.076	0.105
**L84T2F2**	47.5	66.8	0.33%	460	0.63%	1122	1742	0.023	0.106	0.165	0.129	0.188
**L84T2F1**	47.5	66.8	0.33%	460	0.63%	1121	1742	0.023	0.106	0.165	0.129	0.188
**L84T1F2**	47.5	66.8	0.33%	460	0.32%	1166	1742	0.023	0.055	0.083	0.078	0.105
**L84T1F1**	47.5	66.8	0.33%	460	0.32%	1167	1742	0.023	0.055	0.083	0.078	0.105

#### 3.2.1. Cracking, Yield, and Ultimate Moments of Tested Members

Table 6 contains results describing the behavior of members during tests. In the second column, values of bending moment in the mid-span are presented when a lowering of member stiffness was observed. It can be detected in the chart of the bending moment–deflection relationship where the diagram transitions from linear to non-linear state. The third column contains values of the bending moment when the first cracks were observed on the member side surfaces. The values presented in the second and third columns of the above-mentioned table can be treated as the lower and upper limits of member cracking.

The fourth column consists of bending moment values when the yielding of the bottom rebars occurred. This was determined by strain observations in the bottom reinforcing bars through the use of electrical strain gauges (TS 3 and TS 4—Figure 12).

The analysis of the ultimate bending moment and the stress increment in unbonded tendons was more complicated than expected. The following factors had an influence on the above parameters: the prestressing reinforcement ratio, span-to-depth ratio, and type of loading.

It was hypothesized that in pairs of elements with one and two tendons (L36T2F2–L36T1F2, L36T2F1–L36T1F1, etc.), a greater value of stress increment in unbonded tendons would be achieved in beams with a smaller reinforcement ratio. This statement was true for all elements except two (L36T2F1–L36T1F1), where the stress increment in members prestressed with a single tendon was smaller.

The hypothesis posited that within groups consisting of three members with equivalent tendons and uniform loading conditions, yet differing span-to-depth ratios (e.g., L36T2F2–L60T2F2–L84T2F2), the highest value of stress increment and bending resistance would be achieved in the shortest members, and the lowest value of stress increment and bending resistance would be gained in the longest members. The statement regarding stress increment was true for one group of three (L36T1F1–L60T1F1–L84T1F1). However, this was not reflected in bending resistance. In other cases, the smallest stress increases in unbonded tendons were obtained in members for which the span length was 6.0 m (group L60), and the greatest stress increase was seen in the two members with a span length of 3.6 m (L36T2F1 and L36T1F2) and one member with a span length of 8.4 m (L84T2F2). The expected decrease in bending resistance was fulfilled only for three members (L36T2F1–L60T2F1–L84T2F1). In other cases, once again, the worst results were observed for beams with a 6.0 m span length (group L60), and the highest values of ultimate bending moment were seen for two members with span lengths of 3.6 m (L36T2F2 and L36T2F1) and one member of 8.4 m (L84T1F2). It should be emphasized that a correlation occurred between high values of stress increment in the tendons and a high bending moment resistance (L36T2F2, L36T2F1, L36T1F2, L36T1F1, L84T2F2, and L84T1F2). Members under third-point loading with span lengths of 3.6 and 8.4 m differed slightly with regard to the stress increments in tendons and ultimate bending moment.

It was hypothesized that, under center-point and third-point loading, the stress increase in prestressing steel and the bending moment resistance would be greater for the latter. This was observed in all members except one pair (L36T2F2 and L36T2F1).

**Table 6:** Cracking, yield, and ultimate moments of tested members.

	Member Stiffness Decrease [kNm]	First Cracking [kNm]	Yield Moment [kNm]	Bending Resistance [kNm]	Stress Increment [MPa]	Compressive Zone Depth [mm]	Compressive Strain [‰]	Compressive Zone Depth [mm]	Compressive Strain [‰]
						1/3 Span Length		1/2 Span Length	
**L36T2F2**	35	40	68	85.3	431	35	2.3	48	1.9
**L36T2F1**	35	35	68	86.4	447	111	0.6	-	3
**L36T1F2**	22	25	39	54.8	575	18	2.8	31	2.6
**L36T1F1**	21	23	45	58.7	343	102	0.4	19	3.2
**L60T2F2**	33	33	65	76.2	312	37	2.3	42	1.9
**L60T2F1**	33	33	65	77.1	183	115	0.6	22	3.4
**L60T1F2**	21	22	40	50.9	474	33/23	1.8/2	40/30	2.1/3.9
**L60T1F1**	24	22	40	48.4	286	100	0.5	25	1.7
**L84T2F2**	33	35	64	83.4	447	41	2.3	38	2.7
**L84T2F1**	33	36	62	74.2	196	114	0.6	-	2.5
**L84T1F2**	24	24	41	56.3	513	21	2.2	21	2.5
**L84T1F1**	23	26	42	50.7	247	71	0.6	21	2.5
**Average T2**	**34**	**35**	**65**						
**Average T1**	**23**	**24**	**41**						

For all members from group L36 and two members from group L60 (L60T2F2 and L60T2F1), bending resistance was found to be greater for members under center-point loading. This may be connected with concrete confinement, which was caused by stirrups and the steel plate used to transfer loading from the hydraulic machine piston. This phenomenon could also be confirmed by the ultimate strain, which is presented in the last column of Table 6. Both the concrete stress block depth and the strain in the top fiber were obtained by extrapolating the strain obtained from electrical strain gauges TC 1, TC2, TC 5, and TC 6 (Figure 12) from the strain diagram (e.g., Figure 18). Due to a failure of the strain gauges, the ultimate strain for L36T2F1, as indicated in Table 6, is not the value at ultimate but rather the last available value. The side surfaces in the mid-span for L60T1F1 were in bad condition, which did not allow the gauges to be properly glued. The presented values are from the cross-section placed 10 cm from the center.

#### 3.2.2. Stress Increase in Unbonded Tendons

As illustrated in Table 7, the behavior of prestressing force is presented, ranging from prestressing to member failure. In the field of engineering calculations, it is generally accepted that the stress increment in unbonded tendons is negligible until the yield of ordinary reinforcement is reached. Predictive equations consider only the stress increment at ultimate. For EC2 (Equation (1a)), the stress increment has a constant value of 100 MPa. For ACI 318, the value can be estimated through the utilization of Equations (2) or (3). These equations consider the span-to-depth ratio. According to ACI 318, the following values of stress increments in unbonded tendons are obtained for tested members:

- For group L36 (span-to-depth ratio smaller than 35): 176 MPa for L36T2F2 and L36T2F1, and 281 MPa for L36T1F2 and L36T1F1.
- For group L60 and L84 (span-to-depth ratio greater than 35): 105 MPa for L60T2F2, L60T2F1, L84T2F2, and L84T2F1, and 140 MPa for L60T1F2, L60T1F1, L84T1F2, and L84T1F1.

The stress increment was found to be significantly higher than the stress predicted by the code equations.

**Table 7:** Stress increases in unbonded tendons in different phases of loading.

	Prestressing Force During Tensioning (Member in Mould) [kN]	Prestressing Force After Wedge Draw-in (Member in Mould) [kN]	Prestressing Force After Initial Relaxation (Test Stand) [kN]	Prestressing Force at First Cracking [kN]	Prestressing Force at Yield of Rebars [kN]	Prestressing Force at Failure [kN]	Stresses After Losses [MPa]	Stress Increment -Cracking [MPa]	Stress Increment -Yielding [MPa]	Stress Increment -Failure [MPa]	Stresses at Failure [MPa]
	***P*_0_**	***P_m_*_0_**	***P_mt_***	***P_mt_***	***P_mt_***	***P_mt_***	***σ_Pmt_***	***Δσ_cr_***	***Δσ_pl_***	***Δσ_ult_***	***σ_Pmt_***
**L36T2F2**	401.2	345.0	340.5	342.6	381.8	470.7	1127	7	137	431	1559
**L36T2F1**	398.5	338.8	335.0	335.9	351.3	469.9	1109	3	54	447	1556
**L36T1F2**	198.5	166.7	167.4	168.3	181.8	254.2	1109	6	95	575	1683
**L36T1F1**	200.7	169.0	171.8	172.1	179.8	223.7	1138	2	53	343	1481
**L60T2F2**	384.9	352.2	340.5	341.8	377.4	434.7	1127	4	122	312	1439
**L60T2F1**	392.5	350.1	343.2	343.6	356.6	398.6	1136	1	44	183	1320
**L60T1F2**	192.2	172.1	170.6	171.0	187.2	242.2	1129	3	110	474	1604
**L60T1F1**	193.5	171.9	171.4	171.6	178.6	214.6	1135	1	47	286	1421
**L84T2F2**	373.0	342.1	338.9	340.2	375.0	473.9	1122	4	120	447	1569
**L84T2F1**	373.1	341.7	338.6	338.9	351.8	397.7	1121	1	44	196	1317
**L84T1F2**	194.6	177.6	176.0	176.2	192.9	253.4	1166	1	112	513	1678
**L84T1F1**	194.6	177.6	176.2	176.3	186.3	213.4	1167	1	67	247	1413

The data gathered in Table 7 could be divided into several phases. The first phase was two-sided prestressing. During this phase, the elastic deformation of concrete occurred. The prestressing force for each tendon was about 200 kN and 190–195 kN for groups L36, and L60 and L84, respectively. During the second stage, the prestressing force was transmitted to the members. The value of prestressing force was reduced due to wedge slip in the anchorage: ~30 kN, ~20 kN, and ~16 kN per tendon for groups L36, L60, and L84, respectively. In the third stage, each member was moved from the mould to the test stand. The value of prestressing force was increased due to the fact that the self-weight of the member started acting and was reduced by the initial relaxation in the strands. The stress in tendons for all members after losses ranged from 1109 to 1167 MPa, with a mean value equal to 1132 MPa. The fourth phase was connected to the occurrence of the first crack at ~35 and ~24 kNm for groups T2 and T1, respectively. The stress increase in the tendons was almost unnoticeable and did not exceed 7 MPa. It could be observed that the stress increment due to cracking was greater in members subjected to third-point loading. The fifth phase was connected to the yielding of rebars, which occurred at a value of the mid-span moment equal to ~65 and ~41 kNm for groups T2 and T1, respectively. As before, the stress increase was related to the type of loading. The mean value of the stress increase in the tendons was ~116 and ~52 MPa for groups F2 and F1, respectively. The stress increase at ultimate was described in a previous point of this article.

#### 3.2.3. Deflection

Table 8 presents the mid-span deflection and the deflection–span length ratio in different phases of loading.

**Table 8:** Deflections of members in different phases of loading.

	**Deflection Due to Self-Weight** **and Prestress**		**Deflection** **at First Cracking**		**Deflection** **at Yield of Rebars**		**Deflection** **at Failure**		**Value of Bending Moment** **at Reaching SLS**			
	**u** **[mm]**	**u/l_eff_** **[-]**	**u** **[mm]**	**u/l_eff_** **[-]**	**u** **[mm]**	**u/l_eff_** **[-]**	**u** **[mm]**	**u/l_eff_** **[-]**	**u_lim_** **[mm]**	**M_Ed_** **[kNm]**	**M_ult_** **[kNm]**	**M_Ed_** **/M_ult_**
**L36T2F2**	−2.6	1/1385	4.0	1/900	24.8	1/145	73.6	1/49	14.4	56.4	85.3	66%
**L36T2F1**	−2.5	1/1440	2.0	1/1800	12.5	1/288	90.2	1/40		69.4	86.4	80%
**L36T1F2**	−1.1	1/3273	3.4	1/1059	16.8	1/214	111.6	1/32		36.6	54.8	67%
**L36T1F1**	−1.1	1/3273	1.9	1/1895	11.4	1/316	58.9	1/61		46.7	58.7	80%
**L60T2F2**	−5.4	1/1111	6.6	1/909	57.0	1/105	133.6	1/45	24.0	53.5	76.2	70%
**L60T2F1**	−5.5	1/1091	3.9	1/1550	27.5	1/218	87.1	1/69		63.0	77.1	82%
**L60T1F2**	−1.4	1/4286	6.7	1/896	47.7	1/126	202.9	1/30		34.4	50.9	68%
**L60T1F1**	−1.4	1/4286	6.5	1/923	28.9	1/208	129.5	1/46		38.3	48.4	79%
**L84T2F2**	−5.6	1/1500	10.5	1/798	106.0	1/79	371.6	1/23	33.6	49.1	83.4	59%
**L84T2F1**	−5.6	1/1500	8.3	1/1012	52.2	1/161	166.4	1/50		55.5	74.2	75%
**L84T1F2**	2.0	1/4200	10.1	1/832	92.0	1/91	501.5	1/17		33.6	56.3	60%
**L84T1F1**	2.0	1/4200	9.5	1/884	65.1	1/129	211.4	1/40		36.8	50.7	73%

**Figure 19:**
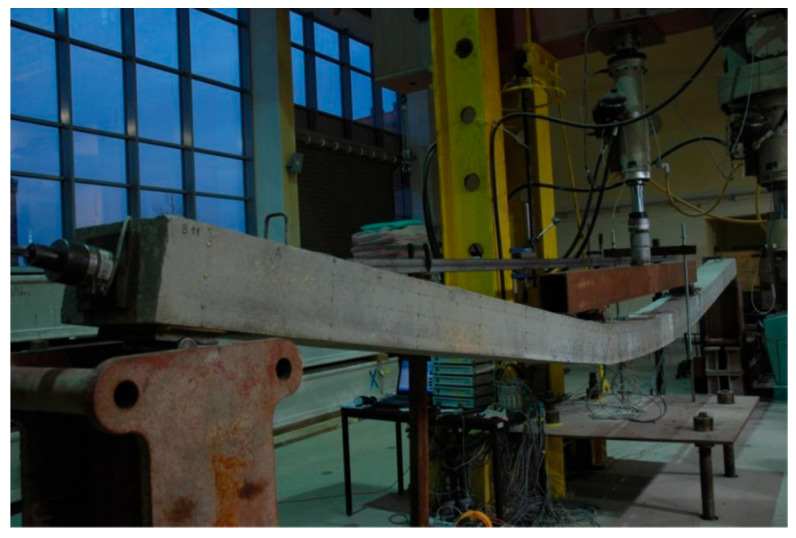
Deflection of L84T1F2 during the last final of loading.

In the second column, there are gathered theoretical (calculated, not measured) deflections resulting from self-weight and prestressing. The third column contains deflections when the first crack occurred: ~35 and ~24 kNm for groups T2 and T1, respectively. These deflections were calculated as the sum of theoretical deflections occurring as a result of self-weight and prestressing and deflections measured during testing. The same procedure was used for the fourth column, where deflections occurring during the yielding of rebars are presented (~65 and ~41 kNm for group T2 and T1, respectively). In the next column are gathered deflections at ultimate, which were obtained in the same manner as described above. The final column, which pertains to deflection, contains the limit value of deflection for each respective group, which corresponds to 14.4 mm, 24 mm, and 33.6 mm for L36, L60, and L84, respectively. The final three columns present the following data: the value of the bending moment at which the SLS relating to the deflection is met (*u_lim_*); the ultimate bending moment; and the ratio of these moments expressed in percentages. As illustrated in Figure 19, the L84T1F2 member exhibited a deflection that exceeded 0.5 m at failure.

#### 3.2.4. Crack Pattern

As illustrated in Table 9, the results obtained from the examination of cracks in the tested members were collected prior to the final phase of loading. The collected dataset includes the following metrics: the length of the cracked region, measured from the first to the last crack; the length of the cracked region relative to the span length; the number of cracks; and the mean distance between cracks.

**Table 9:** Crack pattern characteristics of tested members.

	L36 T2F2	L36 T2F1	L36 T1F2	L36 T1F1	L60 T2F2	L60 T2F1	L60 T1F2	L60 T1F1	L84 T2F2	L84 T2F1	L84 T1F2	L84 T1F1
**Length of cracked region**	1800	825	1630	900	3140	1900	3620	1900	4900	2220	5050	3340
[mm]												
**Cracked region length to span length ratio**	50%	23%	45%	25%	52%	32%	60%	32%	58%	26%	60%	40%
[-]												
**Number of cracks**	14	7	12	7	23	14	32	13	38	17	39	22
[-]												
**Average crack spacing**	138	138	148	150	143	146	117	158	132	139	133	159
[mm]												

A correlation between the observed crack pattern and the type of loading was identified. It is evident that the magnitude of this phenomenon is amplified among members of group F2, where the length of the constant moment region is equivalent to one-third of the span length. It is noteworthy that for members of group F1, the cracked region did not exclusively encompass the proximity of the mid-span. The ratio of the cracked region length to the span length was minimal for L36T2F1 and maximal for L84T1F1, with values of 23% and 40%, respectively. The range of values for the ratio of the cracked region length to the span length for members in group F2 was found to vary from 45% to 60%. The minimum value was obtained for member L36T1F2, while the maximum was obtained for members L60T1F2 and L84T1F2. It has been observed that the value of this ratio increases with the span length. This phenomenon can be attributed to the fact that, for members with a greater span length, the participation of the bending moment caused by self-weight (uniformly distributed loading) in the ultimate moment is greater. Additionally, a correlation between the observed crack pattern and the stirrup spacing, which was 150 mm, was noted. The mean values of crack spacing were found to be nearly equivalent to this value for all members. Further cracks were detected in the vicinity of point loading. In the final phase of loading, stirrups were discernible through the cracks, exhibiting a width of several millimeters. The primary conclusion concerning cracks is that the implementation of longitudinal rebars that met EC2 [3] requirements, even with a low prestressing reinforcement ratio, provided a satisfactory crack pattern and exhibited appropriate behavior in the tested members.

## 4. Final Conclusions

The present study investigates the behavior of twelve beams that have been post-tensioned with unbonded tendons. The issue of stress increments in prestressing reinforcement should not be overlooked or simplified in the analysis of the ULS and SLS of those types of structures. The results of laboratory tests and numerical analyses allowed the formulation of the following conclusions:

- The reinforcement ratio for deformed bars was found to be in accordance with the requirements delineated in both EC2 [3] and the aforementioned literature. This approach ensured the formation and propagation of cracks in a controlled manner. This approach also enabled the consideration of member behavior as beams rather than as a truss model. The mechanical reinforcement ratio, designated as q_0_, for elements from both groups T1 and T2, assumed values within the interval where a substantial stress increment in tendons was anticipated. The stress increment in prestressing steel was found to exceed the values obtained from the predicting equations of EC2 [3] and ACI 318 [4].
- The initial value of prestressing force per tendon exhibited a range of 167 to 176 kN. The stress increment in the prestressing strands until the occurrence of the first crack is negligible (but greater for members from group F2 than from F1). A similar observation could be made at the time of deformed bar yielding. The stress increment in the tendons was approximately 100 MPa and 50 MPa for the elements that were tested from groups F2 and F1, respectively. An examination of the data reveals that, for phases preceding the attainment of the yielding moment, the nature of the loading (i.e., the configuration of the bending moment diagram) exerts the most significant influence on the value of the prestressing force. In the instance of failure, the influence of the prestressing reinforcement ratio on the stress increment in tendons could also be observed. For elements with equivalent span lengths and loading types, a greater stress increase was observed for members prestressed with a single tendon (group T1) compared to those with two tendons (group T2). However, this phenomenon was not observed for the two shortest beams under center-point loading (L36T2F1 and L36T2F2), which was likely due to the effects of concrete confinement. The stress levels exhibited an increase from 431 to 575 MPa and from 183 to 286 MPa for beams from groups F2 and F1, respectively. It should be noted that this summary excludes element L60T2F2. It is worth noting that an anomalous auditory phenomenon was detected during the final stage of the loading process. Initially, the condition was identified as a wire rupture in a tendon. Subsequent to the beam testing, the tendon was subjected to a thorough inspection, which revealed that the wires remained intact. Consequently, the noise was likely attributable to tendon slip within the anchorage.
- It has been demonstrated that, owing to minimal stress increments in tendons up to the occurrence of the first crack, the sole parameter exerting an influence on the cracking moment was the prestressing steel ratio. The mean value of the cracking moment for members from group T1 (one tendon) and T2 (two tendons) was approximately 24 kNm and 35 kNm, respectively. As previously indicated, the crack pattern was deemed satisfactory. The extent of the damaged region was contingent on the type of loading and ranged from 23 to 40% and from 45 to 60% of the span length for beams from groups F1 and F2, respectively. It is imperative to acknowledge that the ratio of the cracked region length to the span length undergoes an increase in proportion with the degree of beam slenderness. This phenomenon can be attributed to the observation that members with larger spans exhibited a greater participation of a dead load moment in the ultimate moment. The spacing of the cracks was found to be regular and consistent with the spacing of the stirrups. The initial cracks manifested in the cross-sections where transverse reinforcement had been implemented. It was observed that supplementary cracks typically manifested during the final phase of loading. Dominant cracks were observed in cross-sections where external point loading was acting.
- The tested members exhibited adequate ductility. The deflection-to-span length ratio ranged from 1/69 to 1/17 at ultimate. The code condition for deflection (L/250) was met for beams under center-point loading at approximately 80%, 80%, and 74% of the ultimate moment for groups L36F1, L60F1, and L84F1, respectively. For beams under third-point loading, this provision was met at approximately 67%, 69%, and 60% of the ultimate moment for groups L36F2, L60F2, and L84F2, respectively.
- The analysis of the bending moment resistance proved to be a more complex undertaking than initially anticipated. A correlation was observed between the stress increment and the bending moment capacity. The values of these factors were found to be the lowest among members of group L60, which had an average span length, in comparison to members of groups L36 and L84. Pairs of members under third-point loading (L36T2F2 and L84T2F2, as well as L36T1F2 and L84T1F2) achieved analogous values of both the stress increase in unbonded tendons and bending resistance. Pairs of elements under center-point loading (L36T2F1 and L84T2F1, as well as L36T1F1 and L84T1F1) indicated that both the stress increase in unbonded tendons and bending resistance decreased with an increase in the span-to-depth ratio. The influence of the type of loading could be observed in the last three pairs of tested members (L60T1F2 and L60T1F1, L84T2F2 and L84T2F1, and L84T1F2 and L84T1F1). This effect was not observed in the three other pairs of elements. The stress increase in the unbonded tendons and the bending moment resistance exhibited comparable values for one pair (L36T2F2 and L36T2F1). In two other pairs (L36T1F2 and L36T1F1, as well as L60T2F2 and L60T2F1), the stress increase in the prestressing steel was greater in members under third-point loading. However, the bending capacity was slightly smaller. This phenomenon is likely attributable to the concrete confinement phenomenon.

## Figures and Tables

**Table 3:** Cross-sectional dimensions and reinforcement arrangement.

Name of Member	L36 T2F2		L36 T2F1		L36 T1F2		L36 T1F1		L60 T2F2		L60 T2F1		L60 T1F2		L60 T1F1		L84 T2F2		L84 T2F1		L84 T1F2		L84 T1F1	
**Cross-section**	**1/3**	**1/2**	**1/3**	**1/2**	**1/3**	**1/2**	**1/3**	**1/2**	**1/3**	**1/2**	**1/3**	**1/2**	**1/3**	**1/2**	**1/3**	**1/2**	**1/3**	**1/2**	**1/3**	**1/2**	**1/3**	**1/2**	**1/3**	**1/2**
**Width b**	301	300	300	300	301	299	302	300	296	298	300	298	302	300	298	300	301	300	298	300	300	300	300	300
[mm]																								
**Height h**	201	201	202	201	200	199	200	202	204	200	200	199	201	200	199	200	203	202	202	204	202	204	203	202
[mm]																								
**Concrete** **cover to A_s2_**	26	26	29	25	24	27	27	28	30	25	26	25	28	27	29	24	32	29	30	25	29	29	27	28
[mm]																								
**Distance from A_s1_ to A_s2_**	144	145	139	144	144	142	141	144	143	142	144	141	140	142	143	144	138	142	142	146	140	144	143	142
[mm]																								
**Concrete** **cover to A_s1_**	31	30	34	32	32	30	32	30	31	33	30	33	33	31	27	32	33	31	30	33	33	31	33	32
[mm]																								

**Table 4:** Loading phases of the tested members.

	L36 T2F2	L36 T2F1	L36 T1F2	L36 T1F1	L60 T2F2	L60 T2F1	L60 T1F2	L60 T1F1	L84 T2F2	L84 T2F1	L84 T1F2	L84 T1F1
**Loading phases up to first crack appearance** **-bending moment values** [kNm]		13.5										
	23.4	23.7	15.7	15.5	27.2	25.1	19.0	17.8	30.5	28.9	22.7	20.9
		26.7										
	43.8	43.8	28.2	28.0	45.2	42.4	29.0	28.4	44.5	43.6	28.9	27.8
**Loading phases up to failure** **-bending moment values** [kNm]		50.1	32.5	32.5								
	58.2	55.9	36.6	36.1	53.7	53.6	36.5	34.8	55.0	54.1	35.3	34.1
		61.8	40.0	40.6	62.2	64.9		40.4	63.6	64.6	40.3	40.9
	72.6	72.2	43.4	44.5	68.2	73.1	43.2	44.5	73.1		45.9	44.2
				47.5							49.0	47.9
	85.3	86.4	54.7	58.4	76.2	77.1	50.9	48.4	83.4	74.2	56.3	50.6

## Data Availability

The raw data supporting the conclusions of this article will be made available by the authors on request.
